# A tailored implementation intervention to implement recommendations addressing polypharmacy in multimorbid patients: study protocol of a cluster randomized controlled trial

**DOI:** 10.1186/1745-6215-14-420

**Published:** 2013-12-05

**Authors:** Cornelia Jäger, Tobias Freund, Jost Steinhäuser, Stefanie Joos, Michel Wensing, Joachim Szecsenyi

**Affiliations:** 1 Department of General Practice and Health Services Research, University Hospital Heidelberg, Voßstrasse 2, Heidelberg 69115, Germany; 2 Scientific Institute for Quality of Healthcare, Radboud University, Medical Centre, PO Box 9101, 114 IQ healthcare, 6500 HB Nijmegen, The Netherlands

**Keywords:** Guideline, Implementation, Knowledge transfer, Polypharmacy, Primary care, Tailored intervention

## Abstract

**Background:**

Multimorbid patients frequently receive complex medication regimens and are at higher risk for adverse drug reactions and hospitalisations. Managing patients with polypharmacy is demanding, because it requires coordination of multiple prescribers and intensive monitoring. Three evidence-based recommendations addressing polypharmacy in primary care are structured medication counselling, use of medication lists and medication reviews to avoid potentially inappropriate medication (PIM). Although promising to improve patient outcomes, these recommendations are not well implemented in German routine care. Implementation of guidelines is often hindered by specific “determinants of change”. “Tailored” interventions are designed to specifically address previously identified determinants. This study examines a tailored intervention tto implement the aforementioned recommendations into German primary care practices. This study is part of the European Tailored Interventions for Chronic Diseases project, which aims at contributing knowledge about the methods used for tailoring.

**Methods/Design:**

The study is designed as a cluster randomized controlled trial with primary care practices of general practitioners (GPs) who are organized in quality circles. Quality circles will be the unit of randomization with a 1:1 ratio. Follow-up time is 6 months. GPs and healthcare assistants in the intervention group will receive training on medication management. Each GP will create a tailored concept of how to implement the three recommendations into his/her practice. Evidence-based checklists for medication counselling and medication reviews will be provided for physicians. A tablet PC with an interactive educational tool and information leaflets will be provided for use by patients to inform about the necessity of continuous medication management. Control practices will not receive special training and will provide care as usual. Primary outcome is the degree of implementation of the three recommendations, which will be measured using a prespecified set of indicators. Additionally, the PIM prescription rate, patient activation, patients’ beliefs about medicine, medication adherence and patients’ social support will be measured.

**Discussion:**

This study will contribute knowledge about the feasibility of implementing recommendations for managing patients with polypharmacy in primary care practices. Additionally, this study will contribute knowledge about methods for tailoring of implementation interventions.

**Trial registration:**

Clinicaltrials.gov ISRCTN34664024

## Background

An increasing number of patients have multiple chronic conditions [1]. Multimorbidity is associated with an increased likelihood of complex medication regimens often consisting of five or more different drugs, commonly defined as polypharmacy [2]. With administration of increasing numbers of drugs, the risk of adverse drug reactions (ADRs) increases substantially [3] thereby causing potentially avoidable hospital admissions [4] and preventable deaths [5]. Managing multimorbid patients with polypharmacy is particularly demanding in primary care practices (PCPs), as it requires coordination of multiple prescribers, profound pharmacological knowledge and intense monitoring of patients.

### Recommendations for patients with polypharmacy

An increasing body of literature has been published regarding strategies to address polypharmacy in multimorbid patients [6]. For the Polypharmacy in Multimorbid Patients (PomP) study, three core recommendations derived from the literature have been identified. German guidelines for polypharmacy in primary care, which were published after we made this selection, refer to these recommendations [7].

(1) Recommendation 1/structured medication counseling (SMC): all patients with polypharmacy and additional risk factors for medication problems should receive SMC at least once per year. In addition to medication-related information, SMC comprises a complete inventory of the medications actually taken (a so-called “brown bag review”) and assessment of patient adherence and possible application problems. A separate appointment should be planned for SMC [8].

(2) Recommendation 2/consequent use of medication lists: All patients with polypharmacy should take along an updated, complete (that is, containing all necessary information) and comprehensible medication list [7,9].

(3) Recommendation 3/medication reviews to reduce potentially inappropriate medication (PIM): The appropriateness of a medication can be judged by using explicit criteria (usually drug-to-avoid lists) and implicit criteria (usually checklists). Since 2010, the PRISCUS list has been available in Germany. It lists 82 drugs as potentially inappropriate for use in older patients [10]. The Medication Appropriateness Index (MAI) is a tool to systematically check individual medication regimens to ensure that they meet criteria such as indication, dosage, interactions and applications [11]. It is recommended that physicians, with the aid of such tools, regularly review the medication regimens of patients with polypharmacy [7].

### Current routine care

Implementation of these recommendations would likely improve medication management and the health status of multimorbid patients with polypharmacy [7]. To date, however, these recommendations have not been implemented well in German routine care.

Deficiencies concerning communication between physicians and patients about their medicine are well-known, both at discharge and in ambulatory care [12-14]. Information about medication is usually given only at the time of first prescription, and monitoring of efficacy and ADRs is often insufficient [14]. Patients are dissatisfied with the information provided about possible side effects and feel that there is little chance to discuss their questions and concerns during the consultation [15]. There is evidence that SMC may increase patient satisfaction [16], improve adherence and reduce ADRs and hospitalizations [17].

Several independent studies have shown that there are discrepancies between medication records and actual medication intake in about 75% of cases [18-20] and that 25% of those discrepancies are potentially harmful [21]. In a recent study, it was reported that 40% of primary care patients in Germany reported taking a mean of two drugs of which their general practitioners (GPs) were unaware [22]. Organizational issues (for example, medication records not being updated) were the most frequent cause of differences between medication records and actual medication intake.

An analysis based on the PRISCUS list showed that 25% of the elderly received at least one PIM prescription in 2010 [23]. Medication errors, defined according to the criteria of the MAI, are frequent. An Austrian study examining the medication regimens of 169 patients with polypharmacy in 22 general practices found that, on average, 2.7 medications per patient were not indicated and that 93.5% had taken at least one non-evidence-based medication [24].

### Healthcare system

The challenge of managing patients with polypharmacy is particularly relevant for countries without an established gatekeeping system. Germany has a strong ambulatory specialist care system in addition to primary care. Patients have free choice of doctors and do not have to be registered at any PCP. Therefore, different care providers are able to alter medication regimens without communication with the GP.

In an attempt to strengthen the coordinating role of GPs, some German health insurance policies offer GP-centred care contracts (Hausarztzentrierte Versorgung, or HzV). Patients who are voluntarily enrolled in an HzV contract agree to get a referral from a GP before they contact a specialist. Specialists who voluntarily participate in HzV are obliged to send a report to the GP after each patient contact. GPs participating in the HzV care contract of one German health insurance in one federal state in Germany (“HzV AOK Baden-Württemberg” care contract) [25] are additionally obliged to participate regularly in quality circles (QCs) [26], which comprise small-group meetings of GPs from one geographical region and written feedback on their individual practice patterns. Currently, there are 309 QCs in the German federal state Baden-Württemberg, each comprising on average 10.9 GPs (±5.6).

### Tailored interventions

Implementation of evidence-based practice in healthcare is often hindered by specific barriers and promoted by enablers, also referred to as “determinants of change”. Interventions designed to address previously identified determinants are often referred to as “tailored interventions” [27]. This study is part of the tailored interventions for chronic diseases (TICD) project, which aims to contribute knowledge to gain a better understanding of methods used to tailor implementation interventions in chronic illness care [28]. On the basis of prior work of the TICD project, the aim of the PomP study is to assess the effectiveness of a tailored implementation intervention for polypharmacy in multimorbid patients.

## Methods

### Trial design

**Figure 1:**
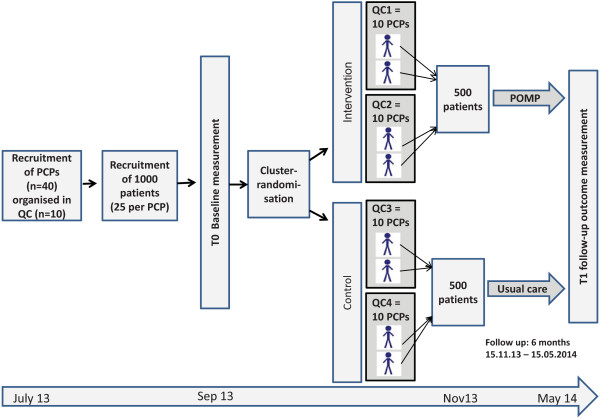
**Trial design of the Polypharmacy in Multimorbid Patients study.** Primary care practices (PCPs) organised in quality circles (QCs) are randomized by QC level. QCs are educational meetings of PCPs from one geographic area.

The intent of the PomP study investigators is to improve the implementation of the aforementioned core recommendations. The aim is to assess the effectiveness of a tailored implementation intervention compared to usual care in professional practice. The study design is a two-armed cluster randomized controlled trial with QCs of PCPs with a 1:1 ratio of units of randomization (see Figure 1). Follow-up time is 6 months.

### Study setting and participants

 PCPs enrolled in the “HzV AoK Baden-Württemberg” care contract and organized in QCs [29] with thrice-monthly meetings will be recruited into the study as participants. Each PCP will include 20 to 25 patients according to the following eligibility criteria.

The eligibility criterion for QCs is the participation of at least 75% of the PCPs of one QC. For PCPs, the criteria for inclusion are enrolment in the HzV AOK Baden-Württemberg care contract and continuous attendance at specific QC meetings every 3 months during the previous 12 months. The eligibility criteria for patients are age older than 64 years, enrolment in the HzV AOK Baden-Württemberg care contract, prescriptions for more than four different drugs in at least one quarter of the year, diagnosis of at least three chronic conditions based on a previously published diagnosis list [30] and high risk of medication problems (according to the personal assessment of the GP, such as nonadherence or hospitalisation due to medication-related events).

Exclusion criteria are PCPs that have participated in another study focused on multimorbidity or polypharmacy during the previous year, low risk of medication problems (as assessed by GPs via chart review) and cognitive or clinical status of patients that hinders active participation in the study.

### Intervention study group

**Figure 2:**
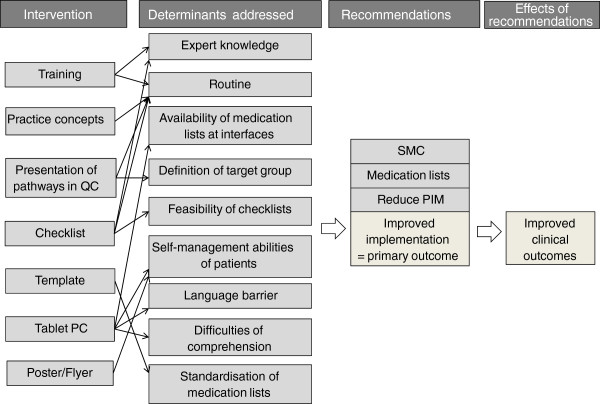
**Logic model of the tailored implementation intervention in the Polypharmacy in Multimorbid Patients study.** QC, quality circle; SMC, structured medication counselling.

The PomP study tailored intervention was developed on the basis of previously identified determinants and strategies. The result of this previous work was a standardised list of determinants for implementing into practice the three core recommendations (SMC, use of medication lists and medication reviews to reduce PIM), as well as a set of strategies addressing these determinants. An implementation intervention has been designed on the basis of this prior work. Each strategy within the implementation intervention addresses one or more specific determinants. Figure 2 specifies which determinant is meant to be addressed by each strategy. The strategies used in the implementation interventions are outlined in the following subsections.

#### Workshops

GPs and healthcare assistants (HCAs) will receive training in four main areas: (1) medication counselling; (2) medication management, including the use of medication lists; (3) pharmacological issues, including PIMs; and (4) organisational study issues, such as documentation, use of tablet PCs and creation of practice-based pathways. A half-day training session will be organised by the study team for all PCPs in the intervention group. At least one GP and one HCA from each PCP must be present. The aim of the training sessions is to increase expert knowledge of medication management.

#### Individual practice concepts

Each PCP team will create an individual concept which describes how they plan to implement the recommendations into their practice. The aim of individual practice concepts is to enhance awareness of the processes and possible performance gaps in the individual practice and to optimise management processes of the PCP by clearly defining responsibilities and tasks and by designing tailored strategies for each PCP.

#### Presentation of individual practice concepts at quality circle meetings

Each QC is obliged to organise one meeting in addition to their regular agenda, during which each practice team will present its individual implementation concept. The aim of this meeting is to increase the likelihood that practice concepts will be elaborated and to set the stage for the exchange of ideas for individual solutions among the QC participants.

#### Checklist for SMC and medication reviews

A checklist for SMC and medication reviews [8] will be handed out to all PCPs of the intervention group and should be used to document each SMC review and each medication review. The checklist for medication reviews will be based on the components of the MAI [11]. The aim of the checklist is to structure and thus facilitate the counselling and review process and to increase routine and quality.

#### Template for medication lists

A template for medication lists will be given to each PCP as an adaptable text file [9]. Practice teams are supposed to make sure that the medication lists they use by default contain the information specified in the template.

#### Tablet PCs for patients

A tablet PC with an interactive educational tool for the three core recommendations for patients will be provided (one tablet PC per PCP). The tool will be available in German, English and Turkish to reach a broader spectrum of patients, including non-German-speaking patients. All patients in the target group, as defined by the previously created patient register, should complete the educational tool at least once. The aim of the tool is to increase patients’ interest in and awareness of medication-related topics and thus introduce a behaviour change that results in a higher proportion of patients carrying a medication list with them and reporting medication changes and problems proactively to GPs. Difficulties in comprehension due to language barriers will be reduced.

#### Campaign

Posters and flyers for patients with information about the three core recommendations will be provided. Flyers should be placed in the PCP waiting room and handed out to the patients in the target group. Posters should be hung on the walls in the PCP. The aim of the campaign is identical to that of the tablet PC; however, all patients visiting the practices of the intervention group will be exposed to the campaign, whereas the tablet PC tool is to be used only by a sample of patients.

Determinants identified and prioritised, but not addressed by this intervention are cognitively impaired patients, lack of resources (time, financial compensation), multiple information sources, information exchange at interfaces, compatibility of practice software, data security, rebate contracts, alternatives to PIM, discharge medication, geriatric/pharmacological consultations, easy access to PIM and entering medication data into software programs.

### Usual care study group

PCPs in the usual care group will participate in thrice-monthly QCs thematically different from those in the intervention group. They will not receive special training in medication management. These PCPs will continue to deliver care as usual within the framework of the GP-centred care contract. GPs in the control group will be informed about the three recommendations by receiving the information leaflet explaining the PomP study.

### Primary outcome

The primary outcome will be the degree of implementation of the three recommendations measured at the patient level, that is the effectiveness of the implementation program on PCP performance. This will be assessed using a set of indicators. The degree of implementation will be expressed by the number of indicators fulfilled per patient included in the study (see Table 1).

**Table 1:** **Indicators of successful implementation of the core recommendations**^**a**^

**Indicator (per included patient)**	**Yes/No**
Recommendation 1: Structured medication counselling	
SMC was performed at least once according to the checklist	Y/N
BBR has been performed at least once	Y/N
Recommendation 2: Use of medication lists	
Patient’s medication list is concordant with physician’s medication list	Y/N
Medication list is consistent with template	Y/N
Patients carry medication list with them	Y/N
Date on medication list not older than 6 months	Y/N
Recommendation 3: Medication checks to reduce PIM	
Medication review was performed according the checklist/MAI score at least once^b^	Y/N

^a^BBR, brown bag review; MAI, Medication Appropriateness Index; PIM, potentially inappropriate medication; SMC, structured medication counselling. ^b^Samsa *et al*. [11].

### Secondary outcomes

All patients entering the PCP on two randomly selected days will be asked whether they take permanent medication, whether they carry a medication list with them and whether they have received a medication list from their GP. Furthermore, the PIM prescription rate (based on the PRISCUS list [10]) per patient will be measured using insurance claims data.

A number of questionnaires will be used to assess medication-related outcomes in detail on a per-patient level: the Patient Activation Measure [31], the Medication Adherence Report Scale [32], the Beliefs About Medicine Questionnaire [33] and the Social Support Questionnaire [34].

On the basis of previous evidence, it is assumed that implementation of the recommendations will lead to improved health outcomes. However, clinical outcomes will not be determined in this study. In order to describe the sample, and in the context of a comprehensive process evaluation, we will collect sociodemographic data about the selected and unselected participants.

### Sample size

We will include two QCs (about twenty practices) in the intervention group and two QCs (about twenty practices) in the control group. Based on data derived from PIM prescriptions at the patient, practice and QC levels, an intracluster coefficient (ICC) of 0.02 at the practice level and an ICC of 0.0008 at the QC level could be estimated based on assumptions regarding the effect of variance inflation factors from two-level trials [35]. Assuming 20 practices and 3,400 patients per group (PIMs will be assessed for all patients of a practice, regardless of whether they are specifically targeted by the program), a reduction of PIM prescriptions with a standardised effect size of 0.6 could be detected with Crohnbach’s α error of 5% and a β error of 20%. Assuming that PCP performance in the other domains (SMC and medication lists) will not be less than performance on PIM prescriptions, an overall effect size of 0.6 is a reasonable expectation for the primary outcome.

### Statistical methods

Data will be analysed in accordance with the Consolidated Standards of Reporting Trials (CONSORT) statement and its extension for cluster randomized trials [36]. A fully specified statistical analysis plan will be provided prior to the beginning of statistical analyses.

Intention-to-treat as well as per-protocol analyses will be performed. Descriptive statistics will be used to summarise characteristics of both PCPs and patients by using means and 95% confidence intervals for continuous data and absolute numbers for categorical or nominal data. A multilevel modelling approach [37] will be applied to evaluate differences between the intervention group and the control group for all outcomes. This approach will be used to account for the hierarchical structure of the data (that is, patients nested within practices nested within QCs). The effect of the intervention on the primary outcome will be tested at the two-sided significance level of 5%. The result will be presented as the difference between group means with the corresponding 95% confidence interval after adjustment for baseline characteristics. Interim analyses are not planned. Statistical analyses will be carried out using R version 2.15.3 or higher software [38].

### Recruitment

#### Recruitment of primary care practices and quality circles

For recruitment of PCPs, the moderators of all QCs in one large geographic region in Baden-Württemberg (about 11 million inhabitants) will be addressed and informed about the study. Moderators will inform GPs participating in the QCs and ask for the GPs’ written informed consent to participate. At least 75% of the PCPs of one QC must participate in order to be included with the QCs in the study.

#### Recruitment of patients

Each GP will receive a deidentified list of patients assigned to the “HzV AOK Baden-Württemberg” care contract who have received repeated prescriptions for more than four drugs in the previous year, based on insurance claims data. For each patient, information regarding age, gender and the prescribed medication will be provided. GPs will create a patient register by selecting 25 patients from this list who are able to participate in the study and have, from the GPs’ point of view, a high risk for medication problems. GPs will ask patients to give their written informed consent to participate.

#### *Incentives*

Each PCP in the intervention and control groups will receive a tablet PC at the beginning of the study as an incentive to participate. PCPs in the intervention group will additionally receive a financial allowance if they complete the study.

### Randomization

QCs will be randomly allocated to the intervention or control group at a ratio of 1:1. We will perform randomization with numbers generated using R software [38]. Randomization will be performed by a research assistant who will not otherwise be involved in the project. After this step, PCPs will be informed about their assignment by an official letter sent from the study coordinating centre. All participants will be able to decline to participate once they know to which group they have been assigned. Allocation will be concealed until baseline data collection is finished in each centre.

### Blinding

Owing to the nature of the intervention, blinding of the participants will not be possible for this intervention (PCPs). Analysing researchers will be blinded to allocation to minimize bias.

### Data collection methods

Trained study nurses will visit the PCPs twice: at T0 and T1. During the PCP visits, all patients entering the PCP will be asked whether they perceive polypharmacy, whether they carry a medication list with them and whether they have received a medication list from their GP. These data will be collected anonymously by using a tally sheet.

Patient and GP questionnaires will be filled in electronically on the tablet PC in deidentified form. Medication reviews and SMC will be documented by each GP for each patient by using a form on the tablet PC. The data will be stored in a secure central server of the University Hospital Heidelberg. Only project personnel will have access to these data.

GPs will print out or copy the medication lists they have stored in their PCP for each patient at T0 and T1. Additionally, GPs will ask patients to show the medication lists they have with them when the patients give their informed consent (T0) and at T1, copy and deidentify these lists and send them to the study team. If a patient does not have a medication list with him or her, a blank form will be sent.

### Ethics

This study was approved by the ethics committee in Heidelberg. The study will be carried out in compliance with the Declaration of Helsinki (2008 version, Seoul, Korea) and local legal regulations. All participants will be asked to provide their written informed consent prior to participation in the study.

## Discussion

In this study, we will evaluate a program designed to improve the implementation of recommendations for patients with polypharmacy in PCP settings. Enrolment in the “HzV AOK Baden-Württemberg” care contract and participation in QCs are inclusion criteria. The participants in this study have therefore different prerequisites for information exchange with other physicians compared to the majority of practices in Germany. However, the impact of this new and still developing care model should not be overestimated. Although the relatively low number of QCs included in this study is not representative, choosing these preexisting organisational structures as settings in this study could be beneficial regarding a wider implementation of the program in the future. Although a performance bias due to the open nature of the trial should be considered, the pragmatic character of the trial implies high external validity and its results are likely to be applicable to other practices in real-life situations. If the implementation program proves to be effective, it could be offered to an established network of about 300 QCs in the region of Baden-Württemberg, thereby contributing to better implementation of important recommendations for managing patients with polypharmacy in PCPs.

## Trial status

Our trial is currently in the planning phase with recruitment of practices started.

## Abbreviations

ADR: Adverse drug reaction; GP: General practitioner; HCA: Healthcare assistant; MAI: Medication appropriateness index; PCP: Primary care practice; PIM: Potentially inappropriate medication; QC: Quality circle; SMC: Structured medication counselling.

## Competing interests

The authors declare that they have no competing interests.

## Authors’ contributions

CJ, TF, JS, SJ and SZ developed the intervention and study protocol. CJ wrote the first draft of the manuscript. TF, SJ, MW and JS critically revised it. All authors read and approved the final manuscript.
